# Distributed and gradual microstructure changes are associated with the emergence of behavioural benefit from memory reactivation

**DOI:** 10.1162/IMAG.a.104

**Published:** 2025-08-22

**Authors:** Martyna Rakowska, Alberto Lazari, Mara Cercignani, Paulina Bagrowska, Heidi Johansen-Berg, Penelope A. Lewis

**Affiliations:** Cardiff University Brain Research Imaging Centre (CUBRIC), School of Psychology, Cardiff University, Cardiff, United Kingdom; Wellcome Centre for Integrative Neuroimaging, FMRIB, Nuffield Department of Clinical Neurosciences, University of Oxford, Oxford, United Kingdom; Experimental Psychopathology Lab, Institute of Psychology, Polish Academy of Sciences, Warsaw, Poland

**Keywords:** targeted memory reactivation, brain plasticity, non-REM sleep, microstructure

## Abstract

Memory reactivation during sleep is known to have an impact upon the gradual consolidation of memory traces, but the nature of plastic changes induced by such reactivation remains unclear. Here, we use diffusion-weighted imaging to track the location and timescale of microstructural changes associated with behavioural effects of Targeted Memory Reactivation (TMR) 20 days post-manipulation, when the behavioural effect first became significant. Because we used a serial reaction time task that is known to draw on the sensorimotor system as well as both medial temporal and striatal memory systems, we included all these areas as regions of interest. We also included precuneus, a structure known for plasticity relating to the neural engram. Our analysis was based on correlations between behavioural benefit of TMR and microstructural plasticity over early (first 24 h) and late (24 h to 10 days) consolidation periods. This showed significant TMR-related microstructural plasticity in the striatum over the early period. Over the late period, we observed TMR-related microstructural changes in both sensorimotor cortex and precuneus. Taken together, these findings demonstrate that TMR-related microstructural changes correlate with subsequent memory benefits across multiple brain regions.

## 1 Introduction

Learning-associated neural processes occurring during sleep have been receiving increasing attention in recent times. The active systems consolidation model suggests that newly encoded memories are reactivated during non-rapid eye movement (NREM) sleep, and that this enables their re-coding from a temporary store to a more permanent location (Born et al., 2006; Born & Wilhelm, 2012; Rasch & Born, 2013). However, while the process of memory consolidation is gradual and occurs over long timescales (Frankland & Bontempi, 2005), it is unclear whether reactivation of memories during sleep leads to structural plasticity over time. Moreover, there is increasing evidence for distributed long-term cortical storage of memories (Dudai, 2004; Josselyn et al., 2015; Josselyn & Tonegawa, 2020), but it is unclear whether replay-driven consolidation is associated with plasticity at different cortical sites. Likewise, how such plasticity could lead to long-term memory storage in humans has not been studied sufficiently (Stee & Peigneux, 2021).

Here, we set out to track the location and timescale of microstructural changes associated with the long-term effects of memory reactivation during sleep using Targeted Memory Reactivation (TMR). TMR involves associating learning items with sensory cues during wake and then covertly re-presenting these cues during sleep (e.g., Rasch et al., 2007; Rudoy et al., 2009). This is thought to trigger reactivation of the cue-associated memory representation which leads to a better recall of the cued items compared to those that were not cued during the night (i.e., uncued) (Antony et al., 2012; Cousins et al., 2014; Rakowska et al., 2021; Schönauer et al., 2014). In recent years, TMR has become a valuable tool to study the mechanisms of sleep-dependent memory processes. It has allowed us to establish a causal link between memory reactivation and consolidation (Belal et al., 2018; Schreiner et al., 2018), and to identify brain regions that are functionally involved in such relationship (Cousins et al., 2016; Rasch et al., 2007; Shanahan et al., 2018; Van Dongen et al., 2012). Our previous study (Rakowska et al., 2021) as well as an independent prior analysis of the current dataset (Rakowska et al., 2024) further demonstrated that the behavioural effects of TMR develop over time and can last up to 3 weeks. However, the structural brain changes driving the long-term functional and behavioural benefits of TMR remain poorly understood. Moreover, it is still unclear whether repeated reactivation of a memory trace can modify tissue microstructure and what the time scale of such changes might be.

**Fig. 1.**
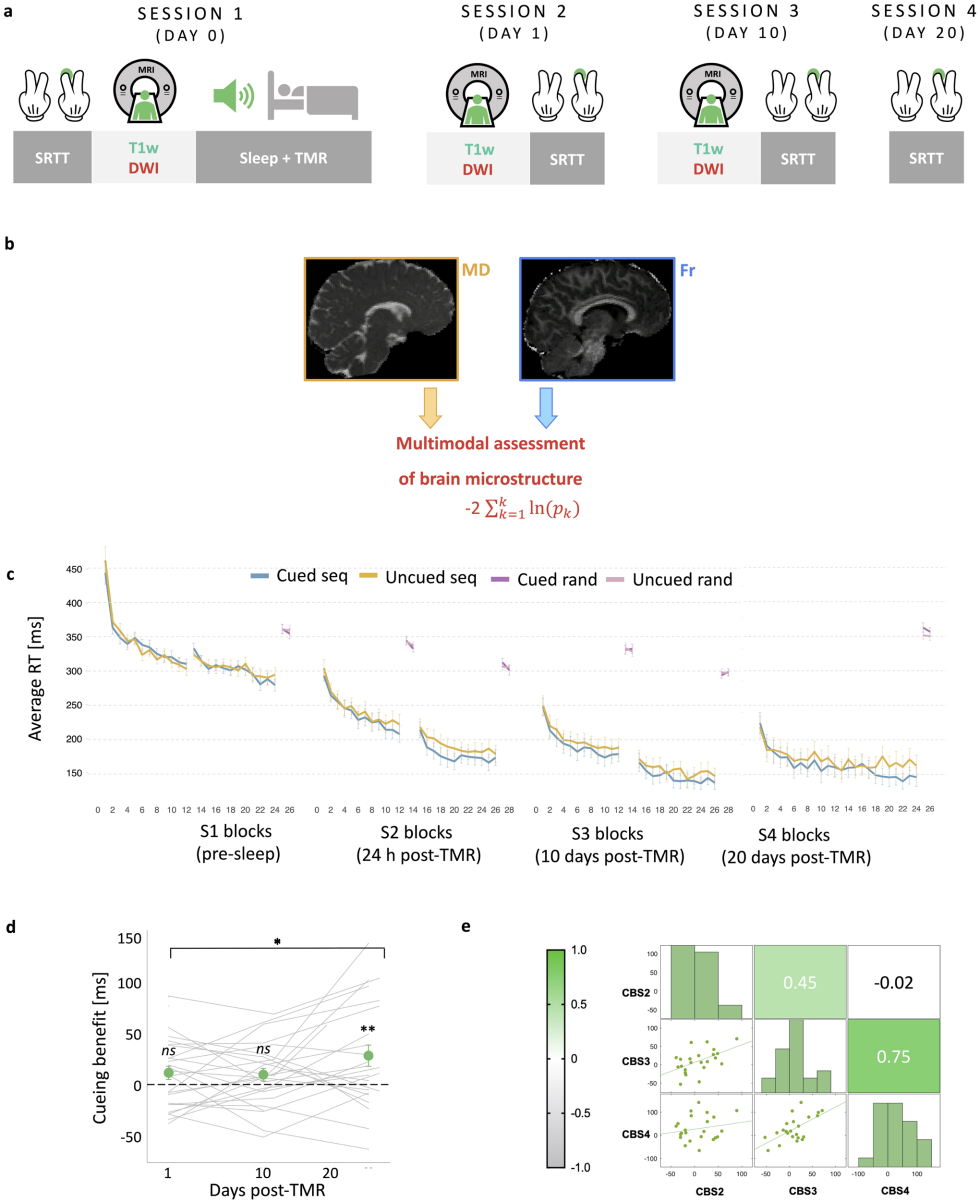
Experimental methods and behavioural effects of TMR. (a) Study design. The study consisted of four sessions, each requiring participants to complete the SRTT. During S1, SRTT was followed by DW-MRI acquisition. During S2 (24 h post-TMR) and S3 (10 days post-TMR), the order was flipped, with the SRTT following MRI data collection. Structural T1w data were acquired at the beginning of each scan (S1-S3). S1 also involved EEG recording during the stimulation night. While asleep, tones associated with one of the sequences were replayed to the participants during stable N2 and N3. During S4 (20 days post-TMR), SRTT was delivered outside the scanner. (b) MRI data analysis. MD and Fr maps were extracted from the DW-MRI data and combined in a joint multi-parameter approach to uncover common trends related to microstructural plasticity of grey matter. The multi-parameter analysis was followed by uni-parameter post-hoc tests to determine the contribution of each parameter to the multi-parameter results (not shown). (c) Average reaction time for the cued sequence (blue), uncued sequence (yellow), and random blocks (light and dark purple) of the SRTT performed before sleep (S1), 24 h post-TMR (S2), 10 days post-TMR (S3), and 20 days post-TMR (S4). Error bars represent the SEM. The data are reported for completeness, for full report see Rakowska et al. (2024). (d) Difference between the late sequence specific skill of the cued and uncued sequence (i.e., the cueing benefit) plotted against the number of days post-TMR. Green dots represent mean ± SEM calculated for S2 (1 day post-TMR), S3 (10–14 days post-TMR), and S4 (16–21 days post-TMR). Grey lines represent cueing benefit for each subject. A linear mixed-effects analysis showed a main effect of time on cueing benefit, which itself was significant at S4. *p < 0.05; **p_adj_ = 0.004. (e) Top: Heatmap of Pearson’s correlation coefficient matrix showing the relationships between cueing benefit at different sessions. Bottom: Scatterplots showing the same correlations. CBS2-S4: cueing benefit at S2-S4; S1-4: Session 1-4; SRTT: Serial Reaction Time Task; DW-MRI: Diffusion Weighted MRI; MD: Mean Diffusivity; Fr: Restricted Water Fraction. For (c): n = 30 for S2, n = 25 for S3; n = 24 for S4. For (d-e): n = 23.

We used Mean Diffusivity (MD) and Restricted Water Fraction (Fr) to examine short- and long-term microstructural plasticity after TMR of a procedural memory task (Fig. 1a). Both MD and Fr are task-independent structural measures which allow probing of the microstructural substrate but do not directly reflect neuronal activation. These metrics are reliable and comparable across subjects (Salvan et al., 2021; Sexton et al., 2014). Importantly, both measures are sensitive to experience-driven plasticity within memory-related areas (Hofstetter et al., 2013; Sagi et al., 2012; Tavor et al., 2013) and have been used to track the development of the neocortical engram, with the microstructural changes driving gains in behavioural performance (Brodt et al., 2018). MD and Fr are, therefore, excellent metrics for studying the gradual microstructural plasticity associated with memory formation over long timescales.

MD provides indirect information about aspects of cortical microstructure (Mori & Zhang, 2006) such as cell density or size (Sagi et al., 2012). MD has recently been shown to decrease in precuneus in response to a series of repeated learning-retrieval epochs during wake (Brodt et al., 2018), which could be regarded as a proxy of memory reactivation during sleep (Himmer et al., 2019). We, thus, hypothesised that TMR during sleep would also lead to MD changes as a result of plasticity within precuneus. We further expected the motor-related regions to undergo long-term microstructural changes, thereby reflecting their slowly evolving reorganisation (Ganguly & Carmena, 2009; Gao et al., 2018; Kami et al., 1995; Kleim et al., 2004; Matsuzaka et al., 2007).

Finally, despite the importance of tissue microstructure for memory formation (Brodt et al., 2018), it is unclear whether baseline tissue microstructure is predictive of memory encoding capacity. To clarify this, we tested the relationship between baseline brain characteristics and the behavioural benefit of our manipulation, thus adding to our current understanding of the factors that influence the effectiveness of TMR (Hu et al., 2020).

## 2 Methods

### 2.1 Participants

The same sample of 33 healthy volunteers that we reported previously (Rakowska et al., 2024) signed a written informed consent to take part in the study, which had been approved by the Ethics Committee of the School of Psychology at Cardiff University. All participants reported being right-handed, sleeping approximately 8 h per night, having no hearing impairment, normal or corrected to normal vision, and no prior knowledge of the tasks performed upon the start of the study. Regular nappers, smokers, subjects who had travelled across more than two time-zones or engaged in any regular night work during 1 month prior to the experiment were not recruited. Further criteria for exclusion included recent stressful life event(s), regular use of any medication or substance affecting sleep, prior history of drug/alcohol abuse, and neurological, psychological, or sleep disorders. Additionally, participants were asked to abstain from napping, extreme physical exercise, caffeine, alcohol, and other psychologically active food from 24 h prior to each experimental session. We also excluded participants with more than 3 years of musical training in the past 5 years due to a probable link between musical abilities and procedural learning (Anaya et al., 2017; Romano Bergstrom et al., 2012). Participants were screened by a qualified radiographer from Cardiff University to assess their suitability for MRI and signed an MRI screening form prior to each scan.

**Fig. 2.**
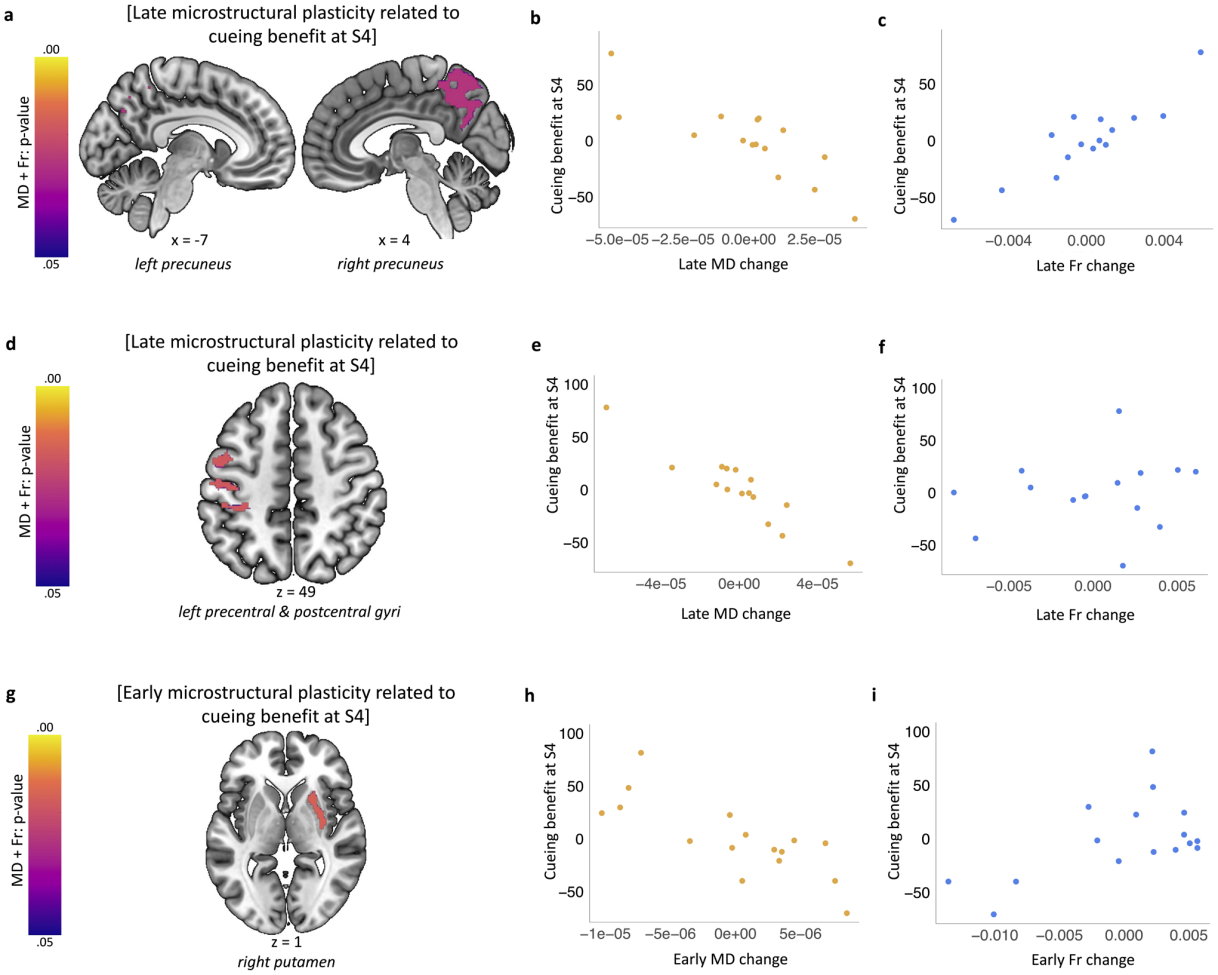
Microstructural plasticity in precuneus and task-related structures is associated with long-term cueing benefits. (a) Early (from S1 to S2) microstructural plasticity in right putamen is associated with cueing benefit at S4. (d) Late (from S2 to S3) microstructural plasticity in bilateral precuneus is associated with cueing benefit at S4. (g) Late (from S2 to S3) microstructural plasticity in left sensorimotor cortex is associated with cueing benefit at S4. Purple-yellow colour bars indicate p-values for the results thresholded at a significance level of p_FWE_ < 0.05, corrected for multiple voxel-wise comparisons within each pre-defined ROI for bilateral putamen (a), precuneus (d), and sensorimotor cortex (g). For multiple voxel-wise comparisons across all four ROIs, see Supplementary Table S2. Results are overlaid on a Montreal Neurological Institute (MNI) brain. (b, c, e, f, h, i) Mean MD (b, e, h) and Fr (c, f, i) change values extracted from joint multi-parameter clusters significant at p_FWE_ < 0.05 shown in (a), (d), and (g). Scatterplots are presented for visualisation purposes only and should not be used for statistical inference. Each data point represents a single participant; axes represent residual values after correcting for age, sex, PSQI score, baseline reaction time, baseline learning capabilities on the SRTT, cueing benefit at S2 and S3. MD: Mean Diffusivity; Fr: Restricted Water Fraction; PSQI: Pittsburgh Sleep Quality Index; S1-S4: Session 1-4; n = 15 for (a-f), n = 16 for (g-i).

Four participants had to be excluded from all analyses due to: technical issues (n = 1), voluntary withdrawal (n = 1), interrupted electroencephalography (EEG) recording during the night (n = 1), and positive slope of learning curve during the first session (indicating lack of sequence learning before sleep) (n = 1). Six additional participants had to be removed from the DW-MRI analyses due to failure of the posterior (n = 2) or anterior (n = 4) part of the radiofrequency coil. Hence, 23 participants remained in the final dataset (12 females, age range: 18–23 years, mean ± SD: 20.42 ± 1.56; 11 males, age range: 19–22 years, mean ± SD: 20.18 ± 0.98). Due to COVID-19 outbreak, four participants were unable to complete the study, missing either one (n = 1) or two (n = 5) sessions. One additional participant could not physically attend S3; they performed the SRTT online, but their MRI data could not be collected and therefore the sample size for the MRI analyses of S3 had to be decreased by one. Finally, Fr maps collected from three additional participants failed a visual quality check after pre-processing and were thus excluded from the Fr analysis (n = 3). Hence, the final sample size for MD analysis was n = 23 for S1, n = 23 for S2 and n = 19 for S3, and the sample size for Fr analysis was n = 20 for S1, n = 20 for S2, and n = 16 for S3. A flowchart of participants included and excluded from the different analyses is presented in Supplementary Figure S3. Note, however, that the different number of participants at different sessions and parameters meant that when these were combined the final sample size had to be decreased. For instance, to create S2-S3 subtraction maps the sample size had to match that of the session with lower number of participants available (i.e., n = 16 for Fr and n = 19 for MD). However, to combine the S2-S3 subtraction maps for a multi-parameter analysis the final sample size for MD had to be decreased by 3 to match the sample size of Fr (i.e., 16 participants). To assess the relationship between late plasticity and cueing benefit at S4 (as in, e.g., Fig. 2d) the sample size had to be further decreased by 1 due to 1 additional participant being unable to attend S4 and hence missing data for the behavioural regressor, leaving only 15 participants available for the analysis.

### 2.2 Study design

The study consisted of four sessions (Fig. 1a), all scheduled for 8 pm to control for the time-of-day effect in MRI data (Trefler et al., 2016). Upon arrival for the first session (S1), participants completed Pittsburgh Sleep Quality Index (PSQI) (Buysse et al., 1989), to examine their sleep quality over the past month. S1 consisted of a motor sequence learning task (the SRTT), MRI data acquisition, and overnight stay in the lab. The SRTT learning session was split in half, such that the first half of the SRTT blocks (24 sequence blocks) was performed in a 0T Siemens ‘mock’ scanner (i.e., an environment that looked exactly like an MRI scanner, but with no magnetic field) and the other half (24 sequence blocks + 4 random blocks) in a 3T Siemens MRI scanner, immediately after T1-weighted (T1w) structural data acquisition. This was followed by DW-MRI (see section 2.6). Participants were then asked to prepare themselves for bed and were fitted with an EEG cap. While in stable stage 2 (N2) or 3 (N3) of NREM sleep, the TMR protocol was initiated (see section 2.5). Briefly, to trigger reactivation of the associated SRTT memories, tones associated with one of the SRTT sequences were replayed to the participants through speakers (Harman/Kardon HK206, Harman/Kardon, Woodbury, NY, USA). Afterwards, on average, 8.81 ± 0.82 h in bed participants were woken up and had the EEG cap removed before leaving the lab.

Session 2 (S2), session 3 (S3), and session 4 (S4) took place 23–26 h, 10–14 days, and 16–21 days after S1, respectively. During S2 and S3, DW-MRI data were acquired as before, followed by an SRTT re-test. Here, the first half of the SRTT blocks (24 sequence blocks + 4 random blocks) was performed in the 3T scanner and the second half (24 sequence blocks + 4 random blocks) in the 0T scanner. Note that the order of scans (3T vs. 0T) was flipped from S1 to S2 and S3 for the microstructural assessment to occur as close to TMR as possible. S4 was performed either in the lab or online, depending on the severity of COVID-19 restrictions at the time. During S4, SRTT was delivered in one run (48 sequence blocks + 4 random blocks).

All experimental tasks were delivered as described before (Rakowska et al., 2024). For offline data collection, the SRTT (S1-S3) was back projected onto a projection screen situated at the end of the MRI/mock scanner and reflected into the participant’s eyes via a mirror mounted on the head coil; during S4 SRTT was presented on a computer screen with resolution 1920 x 1080 pixels and executed using MATLAB 2016b (The MathWorks Inc., Natick, MA, USA) and Cogent 2000 (developed by the Cogent 2000 team at the Functional Imaging Laboratory and the Institute for Cognitive Neuroscience, University College, London, UK). For online data collection (S4), SRTT was programmed in Python using PsychoPy 3.2.2. (Peirce et al., 2019) and administered through Pavlovia online platform (https://pavlovia.org/).

### 2.3 Experimental tasks—the serial reaction time task (SRTT)

The SRTT was used to induce and measure motor sequence learning. It was adapted from (Cousins et al., 2014) and implemented exactly as described before (Rakowska et al., 2024). Briefly, participants learned two 12-item sequences of auditorily and visually cued key presses. The task was to respond to the stimuli as quickly and accurately as possible, using index and middle fingers of both hands. The two sequences—A (1-2-1-4-2-3-4-1-3-2-4-3) and B (2-4-3-2-3-1-4-2-3-1-4-1)—were matched for learning difficulty, did not share strings of more than four items, and contained items that were equally represented (three repetitions of each). Each sequence was paired with a set of 200 ms-long tones, either high (5^th^ octave, A/B/C#/D) or low (4^th^ octave, C/D/E/F) pitched, that were counterbalanced across sequences and participants. For each item/trial, the tone was played with simultaneous presentation of a visual cue in one of the four corners of the screen. Visual cues consisted of neutral faces and objects, appearing in the same location regardless of the sequences (1 – top left corner = male face, 2 – bottom left corner = lamp, 3 – top right corner = female face, 4 – bottom right corner = water tap). Participants were told that the nature of the stimuli (faces/objects) was not relevant for the study. Their task was to press the key on the keyboard (while in the sleep lab or at home) or on an MRI-compatible button pad (2-Hand system, NAtA technologies, Coquitlam, Canada) (while in the MRI/mock scanner) that corresponded to the position of the picture as quickly and accurately as possible: 1 = left shift/left middle finger button; 2 = left Ctrl/left index finger button; 3 = up arrow/right middle finger button; 4 = down arrow/right index finger button. Participants were instructed to use both hands and always keep the same fingers on the appropriate response keys. The visual cue disappeared from the screen only after the correct key was pressed, followed by a 300 ms interval before the next trial. There were 24 blocks of each sequence (a total of 48 sequence blocks per session), where block type was indicated with ‘A’ or ‘B’ displayed in the centre of the screen. Each block contained three sequence repetitions (36 items) and was followed by a 15s pause/break, with reaction time (RT) and error rate feedback. Blocks were interleaved pseudo-randomly with no more than two blocks of the same sequence in a row. Participants were aware that there were two sequences but were not asked to learn them explicitly. Block order and sequence replayed were counterbalanced across participants.

During each run of the SRTT, sequence blocks A and B were followed by 4 random blocks, except for the first half of S1 (to avoid interrupted learning). Random blocks were indicated with ‘R’ appearing centrally on the screen and contained pseudo-randomised sequences, the same visual stimuli, and tones matching sequence A for half of them (Rand_A) and sequence B for the other half (Rand_B). Blocks Rand_A and Rand_B were interleaved, and the random sequences contained within them followed three constraints: (1) each cue was represented equally within a string of 12 items, (2) two consecutive trials could not contain the same cue, and (3) random sequence did not share a string of more than four items with either sequence A or B.

### 2.4 EEG data acquisition

EEG data acquisition was performed exactly as described before (Rakowska et al., 2024). Briefly, EEG was recorded using 64 actiCap slim active electrodes (Brain Products GmbH, Gilching, Germany), with 62 electrodes embedded within an elastic cap (Easycap GmbH, Herrsching, Germany). This included the reference positioned at CPz and ground at AFz. The remaining electrodes were the left and right electrooculography (EOG) electrodes (placed below and above each eye, respectively), and left and right electromyography (EMG) electrodes (placed on the chin). Supplementary Figure S4 shows the EEG electrodes layout. Elefix EEG-electrode paste (Nihon Kohden, Tokyo, Japan) was used for stable electrode attachment, and Super-Visc high viscosity electrolyte gel (Easycap GmbH) was inserted into each electrode to reduce impedance below 25 kOhm. To amplify the signal, we used either two BrainAmp MR plus EEG amplifiers or a LiveAmp wireless amplifier (all from Brain Products GmbH). Signals were recorded using BrainVision Recorder software (Brain Products GmbH).

### 2.5 TMR during NREM sleep

Tones associated with one of the learned sequences (A or B, counterbalanced across participants) were replayed to the participants during N2 and N3, using a protocol described before (Rakowska et al., 2021, 2024). The target sleep stages were assessed with standard AASM criteria (Berry et al., 2015). Volume was adjusted manually for each participant to make sure that the sounds did not wake them up. As in prior papers (Belal et al., 2018; Cousins et al., 2016; Rakowska et al., 2021) to avoid interference between cues, the inter-trial-interval was much longer in sleep than in wake. One repetition of a sequence was followed by a 20 s break, with the inter-trial interval jittered between 2500 and 3500 ms. Upon arousal or leaving the relevant sleep stage, replay was paused immediately and resumed only when stable N2/N3 was apparent. TMR was performed for as long as a minimum threshold of ~1000 trials in N3 was reached. On average, 1552.91 ± 215.00 sounds were delivered. The protocol was executed using MATLAB 2016b and Cogent 2000.

### 2.6 MRI data acquisition

Magnetic resonance imaging (MRI) data were acquired using a 3T Siemens Connectom scanner (maximum gradient strength 300 mT/m) with a 32-channel head-coil. All scans were performed at Cardiff University Brain Imaging Centre (CUBRIC) and lasted ~1 h in total each. This paper is concerned with the analysis of the multi-shell DW-MRI, but the MRI protocol also included T1w, functional MRI (fMRI), and mcDESPOT acquisitions, the analyses of which are reported in separate publications (for T1w and fMRI results, see Rakowska et al. (2024)).

#### 2.6.1 T1-weighted imaging

A high-resolution T1-weighted anatomical scan was acquired with a 3D magnetization-prepared rapid gradient echoes (MPRAGE) sequence as described before (Rakowska et al., 2024) (repetition time [TR] = 2300 ms; echo time [TE] = 2 ms; inversion time [TI] = 857 ms; flip angle [FA] = 9°; bandwidth 230 Hz/Pixel; 256 mm field-of-view [FOV], 256 x 256 voxel matrix size, 1 mm isotropic voxel size; 1 mm slice thickness; 192 sagittal slices; parallel acquisition technique [PAT] with in-plane acceleration factor 2 (GRAPPA); anterior-to-posterior phase-encoding direction; 5 min total acquisition time [AT]) at the beginning of each scanning session.

#### 2.6.2 Multi-shell diffusion-weighted imaging

Diffusion-Weighted MRI data were acquired with a monopolar sequence (TR = 3000 ms; TE = 59 ms; FA = 90°; 266 gradient directions distributed over 6 shells (b = 200, 500, 1200, 2400, 4000, 6000 s/mm^2^); 13 interspersed b = 0 images; bandwidth 2272 Hz/Pixel; 220 mm FOV; 220 x 200 voxel matrix size; 2 mm isotropic voxel size; 2 mm slice thickness; 66 axial-to-coronal slices obtained parallel to the AC–PC line with interleaved slice acquisition; PAT 2 (GRAPPA); multi-band acceleration factor = 2; AT = 14 min) in an anterior-to-posterior phase-encoding direction, with one additional b = 0 posterior-to-anterior volume.

### 2.7 Data analysis

#### 2.7.1 Behavioural data

PSQI global scores were calculated based on the original scoring system (Buysse et al., 1989), and the SRTT analysis was performed as described before (Rakowska et al., 2024). Briefly, the SRTT performance was measured using mean reaction time per block of each sequence (cued and uncued). All trials within each block were considered (i.e., trials performed by both hands), except for those with reaction time exceeding 1000 ms. For each sequence during each session, the mean performance on the last four blocks was subtracted from the mean performance on the two random blocks, thus yielding a measure of late ‘sequence-specific skill’ (SSS). We chose to focus on late SSS rather than early SSS given that our previous study (Rakowska et al., 2021) as well as an independent prior analysis of the current dataset (Rakowska et al., 2024) showed a main effect of TMR on the former only.

To obtain a single measure reflecting the effect of TMR on SRTT performance at each session, we calculated the difference between the late SSS of the cued and uncued sequence and refer to it as the ‘cueing benefit’. Cueing benefits at S2 and S3 were entered as covariates of no interest in the analyses testing the relationship between cueing benefit at S4 and brain microstructure (see section 2.8.2*).*

#### 2.7.2 DW-MRI data pre-processing

A key limitation of MD is that it reflects contributions from all cells within a voxel, as well as extracellular water. To address this, more advanced models have been developed to differentiate between intra- and extracellular water dynamics, such as the Composite Hindered and Restricted Model of Diffusion (CHARMED) (Assaf et al., 2004). By incorporating both hindered and restricted diffusion components, CHARMED enables the estimation of the volume fraction of the restricted compartment (Fr), which has been shown to enhance sensitivity to neuroplasticity (Tavor et al., 2013). Fr primarily reflects the contribution of intracellular cylindrical structures, including axons, dendrites, and glial cell processes. Since dendrites and neural/glial processes are known to undergo structural changes in response to plasticity (Blumenfeld-Katzir et al., 2011; Tavor et al., 2013; Theodosis et al., 2008), this measure offers valuable insights into neurobiological adaptations not only increasing sensitivity to plastic changes, but also facilitating their interpretation. We thus combined MD with Fr in a joint multi-parameter analysis protocol to uncover common microstructural patterns across the two MRI markers and their relationship with TMR benefits in the long term (Fig. 1b).

DW-MRI data pre-processing was performed as described in previous publications (Genc et al., 2020; Tax et al., 2021). The pre-processing steps included (1) Slicewise OutLIer Detection (SOLID) (Sairanen et al., 2018); (2) full Fourier Gibbs ringing correction (Kellner et al., 2016) using Mrtrix mrdegibbs software (Tournier et al., 2012); and (3) a combined topup, eddy, and DISCO step (Rudrapatna et al., 2018) to (i) estimate susceptibility-induced off-resonance field and correct for the resulting distortions using images with reversed phase-encoding directions, (ii) correct for eddy current distortions, and (iii) correct for gradient nonlinearity. To generate Mean Diffusivity (MD) maps, the diffusion tensor model was fitted to the data using the DTIFIT command in FSL for shells with b < 1500 s/mm^2^. To estimate Restricted Water Fraction (Fr) metric, the composite hindered and restricted model of diffusion (CHARMED) was fitted to the data using an in-house non-linear least square fitting algorithm (De Santis et al., 2014) coded in MATLAB 2015a. The two indices (MD, Fr) were chosen based on the existing human literature on the microstructural changes following learning MD: (Brodt et al., 2018; Hofstetter et al., 2013; Sagi et al., 2012; Tavor et al., 2020) Fr: (Tavor et al., 2013). MD describes the average mobility of water molecules and has shown sensitivity to changes in grey matter (Brodt et al., 2018; Sagi et al., 2012; Tavor et al., 2020). MD is thought to reflect the underlying, learning-dependent remodelling of neurons and glia, that is, synaptogenesis, astrocytes activation, and brain-derived neurotrophic factor (BDNF) expression, as confirmed by histological findings (Sagi et al., 2012), which were of particular interest in this study. As opposed to DTI, the CHARMED model separates the contribution of water diffusion from the extra-axonal (hindered) and intra-axonal (restricted) space (Assaf et al., 2004), thereby providing a more sensitive method to look at the microstructural changes than DTI (Tavor et al., 2013). Fr is one of the outputs from the CHARMED framework. In grey matter, Fr changes are thought to reflect remodelling of dendrites and glia and were observed both short-term (2 h) and long-term (1 week) following a spatial navigation task (Tavor et al., 2013).

Co-registration, spatial normalisation, and smoothing of the MD and Fr maps were performed in SPM12, running under MATLAB 2015a. First, we co-registered the pre-processed diffusion images with participants’ structural images using a rigid body model. The co-registration output was then spatially normalised to MNI space. This step involved resampling to 2 mm voxel with B-spline interpolation and utilised T1 deformation fields generated during fMRI analysis of the same participants (Rakowska et al., 2024). That way, the resulting diffusion images were in the same space as the fMRI and T1w data. Finally, the normalised data were smoothed with an 8 mm FWHM Gaussian kernel.

### 2.8 Statistical analysis

All behavioural tests conducted were two-tailed, and both positive and negative contrasts were performed for the MRI analyses. MRI results were voxel-level corrected for multiple comparisons by family wise error (FWE) correction for the whole-brain grey matter (GM) and for the pre-defined anatomical regions of interest (ROI, see section 2.8.4), with the significance threshold set at p_FWE_ < 0.05. To obtain a whole-brain GM mask, the SPM12 tissue probability map of GM was thresholded at 50% probability (Ceccarelli et al., 2012).

#### 2.8.1 Behavioural data

Statistical analyses of behavioural data were conducted in an independent analysis of the same dataset (Rakowska et al., 2024). We used lme4 package (Bates et al., 2015) in R to fit two linear mixed-effects models to our data. The first model (model 1) was used to test the effect of TMR (cued vs. uncued) and Session (S2, S3, S4) on the late SSS. The second model (model 2) was used to test the effect of Time (i.e., number of days post-TMR) on cueing benefit. To account for the repeated-measures design, participant code was always entered as a random intercept.

$$\begin{array}{l} >\;model1\;=\;lmer(lateSSS\sim Session\;+\;TMR\;\\ \;\;\;\;\;\;+\;\left(1|Participant\right),data=dataset) \end{array}$$

$$\begin{array}{l} >\;model2\;=\;lmer(CueingBenefit\sim Time\;\\ \;\;\;\;\;\;+\;\left(1|Participant\right),data=dataset) \end{array}$$

An ANOVA function in R was used to run likelihood ratio tests between the full model and the model without the effect of interest. This allowed us to obtain a p-value for each effect tested. Emmeans package (Lenth et al., 2019) was used to conduct Holm-adjusted post-hoc pairwise comparisons. The results of both the likelihood ratio tests and post-hoc comparisons are cited in this manuscript and discussed in relation to the underlying tissue microstructure.

#### 2.8.2 Joint multi-parameter analysis

Group-level analyses of DW-MRI data were performed in FSL (FMRIB’s Software Library, http://www.fmrib.ox.ac.uk/fsl) (Smith et al., 2004). To examine the relationship between brain characteristics and our variables of interest, we performed non-parametric combination (NPC) for joint interference analysis (Fig. 1b), as described before (Lazari et al., 2021; Sampaio-Baptista et al., 2020). Specifically, NPC was performed over MD and Fr maps to uncover common trends related to non-myelin GM microstructure (Sagi et al., 2012; Tavor et al., 2013).

The analysis was performed through Permutation Analysis of Linear Models (PALM) in FSL (Winkler et al., 2016), using voxel-wise Fisher test with the following equation:

$$-2\;\sum_{k=1}^{k}\;ln\;ln(p_{k})\;$$

where k denotes the total number of parameters being combined, and p_k_ denotes the p-value for a given parameter (Winkler et al., 2016).

NPC Fisher’s combining function tests for effects with concordant directions across parameters of choice. Thus, to test for positive effects across our parameters, imaging data with mismatching directions (here MD) were multiplied by (-1). The significance of the resulting, single joint statistic was assessed through 5000 permutations of each of the separate tests and a cluster-forming threshold of t > 1.75 (equivalent to p < 0.05, based on the degrees of freedom for the smallest sample) at 5% FWE rate. Correction for multiple comparisons was carried out both for the whole-brain GM and for the pre-defined ROIs.

We set out to test for a linear relationship between early (S1 vs. S2) and late (S2 vs. S3) microstructural plasticity and long-term cueing benefit (i.e., at S4). We chose to analyse S1 vs. S2 and S2 vs. S3 separately to look at non-overlapping changes over distinct periods of time. To this end, the images used to assess the longitudinal effects of time were generated by subtracting the pre-processed MD and Fr parameter maps from consecutive sessions. The resultant images were entered into a one-sample t-test with cueing benefit at S4 added as a main regressor and the remaining sessions (S2 and S3 where no behavioral benefit was apparent), treated as the covariates of no interest (nuisance covariates) to control for inter-session variations in behavioural trajectories that are unrelated to emergence of the cueing benefit in S4. This ensured that the results were specific to the session analysed. Additionally, the nuisance covariates also included sex and age to control for the differences between males and females, as well as the effect of age. Baseline reaction time (i.e., average reaction time on the random blocks performed during S1) and baseline learning capabilities (i.e., difference between the average of the last four blocks and the first four blocks performed during S1) were also specified as the variables of no interest to ensure that the results were independent of baseline SRTT performance.

To determine whether individual differences in baseline brain characteristics can predict susceptibility to the manipulation, we tested the relationship between baseline (S1) GM microstructure and cueing benefit at S4. Thus, cueing benefit at S4 was entered as a covariate of interest in a one-sample t-test within the joint multi-parameter framework. The nuisance covariates for this analysis included: cueing benefit at S2 and S3, sex, age, PSQI, baseline reaction time, baseline learning capabilities, and percentage of time spent in N2 (given the results described in Rakowska et al., 2024). This approach ensured that the results were independent of demographics, general sleep patterns, and baseline SRTT performance, all of which could be related to baseline characteristics of the brain.

#### 2.8.3 UNI-parameter analysis

To determine individual contribution of each microstructural parameter to the multi-parameter results, we performed uni-parameter analyses of individual parameters, in FSL and through non-parametric, permutation-based voxel-wise comparisons using the *randomise* function (Winkler et al., 2014). Results were derived from 5000 permutations. Correction for multiple comparisons was carried out by FWE correction both for the whole-brain GM and for the pre-defined ROIs, as for the multi-parameter analysis. Multiple parameters correction was performed based on the number of parameters entered in the multi-parameter analysis (here two: MD and Fr).

#### 2.8.4 Regions of interest

All MRI results were voxel-level corrected for multiple comparisons within the whole-brain GM and the pre-defined anatomical ROIs. Our pre-defined ROIs included (1) bilateral precuneus, (2) bilateral dorsal striatum (putamen and caudate), (3) bilateral hippocampus and parahippocampus, and (4) bilateral sensorimotor cortex (precentral and postcentral gyri). All ROIs were selected based on their known involvement in sleep-dependent procedural memory consolidation (Albouy et al., 2008; Debas et al., 2010; Fischer et al., 2005; Walker et al., 2005) and memory reactivation (Brodt et al., 2018; Cousins et al., 2016; Maquet et al., 2000, 2003; Rasch et al., 2007; Van Dongen et al., 2012). Any contrast that yielded significant results for either one of our pre-defined ROIs was re-entered into the analysis and corrected for multiple comparisons within a single mask combining all the pre-defined ROIs, thus accounting for multiple small volume corrections. Wake Forest University (WFU) PickAtlas toolbox was used to create a mask for each ROI, based on an Automated Anatomical Labeling (AAL) atlas (Maldjian et al., 2003).

### 2.9 Results preparation

**Fig. 3.**
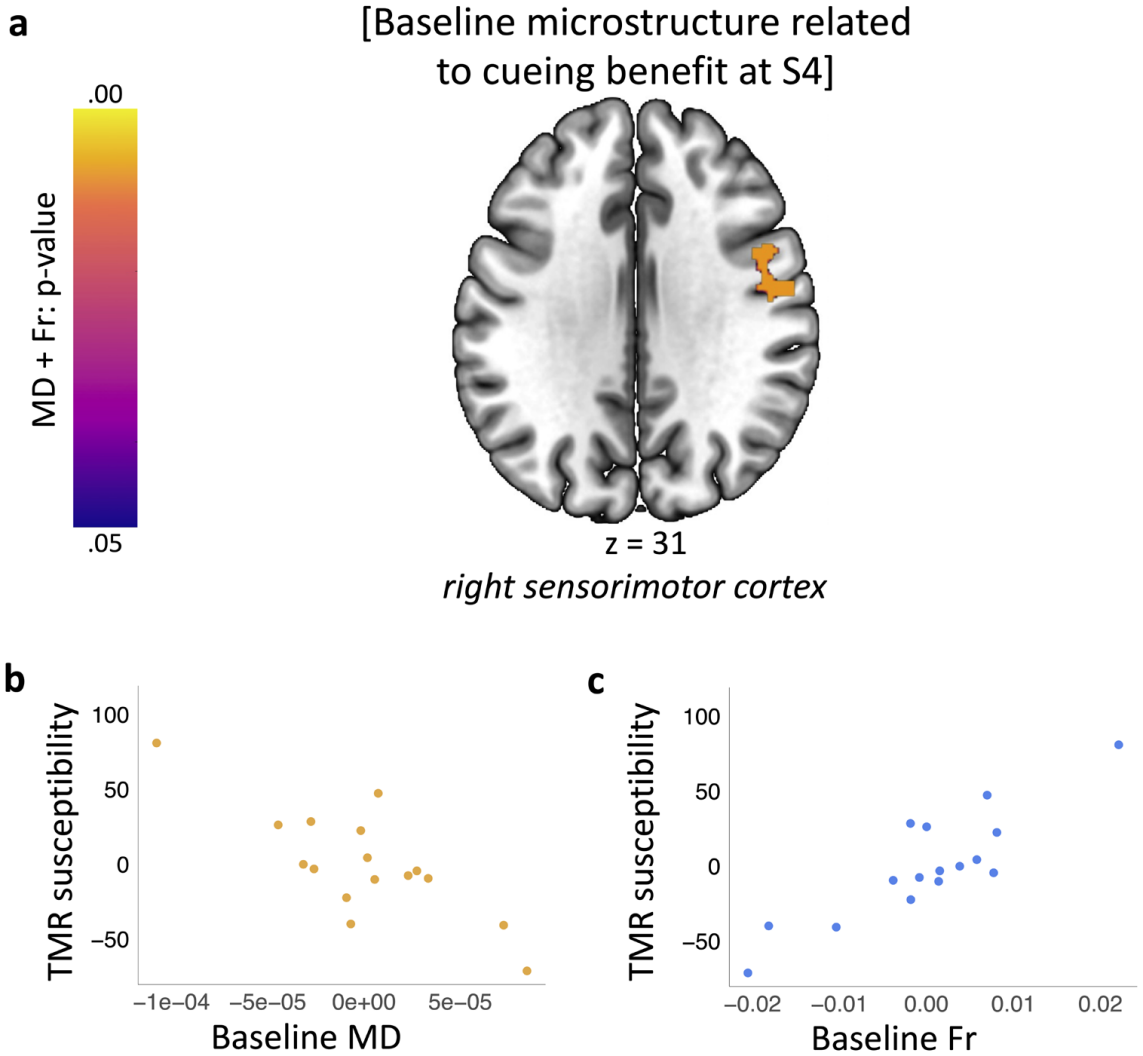
Cueing benefit at S4 is associated with baseline sensorimotor microstructure. (a) Results of the joint multi-parameter analysis testing the relationship between cueing benefit at S4 and baseline microstructure. Colour bar indicates p-values for the results thresholded at a significance level of p_FWE_ < 0.05 (red), corrected for multiple voxel-wise comparisons within pre-defined ROI for bilateral sensorimotor cortex. Results are overlaid on a Montreal Neurological Institute (MNI) brain. (b, c) Mean baseline MD (b) and Fr (c) extracted from the clusters significant at p_FWE_ < 0.05 shown in (a). Scatterplots are presented for visualisation purposes only and should not be used for statistical inference. Each data point represents a single participant; axes represent residual values after correcting for cueing benefit at S2 and S3, age, sex, PSQI score, percentage of time spent in N2, baseline reaction time, and baseline learning capabilities on the SRTT. MD: Mean Diffusivity; Fr: Restricted Water Fraction; PSQI: Pittsburgh Sleep Quality Index; S1-4: Session 1-4; n = 16.

Anatomical localisation of the significant clusters from both uniparameter and multi-parameter analyses was determined with the automatic labelling of MRIcroGL (https://www.nitrc.org/projects/mricrogl/) based on the AAL atlas. Results in Figures 2 and 3 and Supplementary Figure S1 and Supplementary Figure S2 are presented using MRIcroGL, displayed on the MNI152 standard brain (University of South Carolina, Columbia, SC). Cluster statistics for all significant clusters are reported in supplementary tables; peak voxel MNI coordinates are given in text. Figure 1, Supplementary Figure S3 and Supplementary Figure S4 were created in Microsoft PowerPoint v16.53; Figure 1d was generated using *ggplot2* (version 3.3.0) (Wickham, 2009) in R; and Figure 1e was generated using Prism 9 (GraphPad Software, San Diego, CA, USA), and *corrplot* command in MATLAB.

## 3 Results

Our participants learned two motor sequences of a Serial Reaction Time Task (SRTT), each associated with a different set of auditory tones (Supplementary Notes: Baseline SRTT performance). Tones associated with one of the sequences were replayed to the participants during subsequent NREM on a single night (see Supplementary Table S5 for sleep parameters). The post-sleep SRTT re-test sessions took place 24.67 h (SD: 0.70) (Session 2, S2), 10.48 days (SD: 0.92) (Session 3, S3), and 20.08 days (SD: 0.97) (Session 4, S4) after Session 1 (S1), with the MRI data acquired before sleep, at S2, and at S3. Raw behavioural data are presented in Figure 1c*.* A prior analysis of the data (Rakowska et al., 2024) showed a main effect of TMR on the SRTT reaction time performance (p = 0.001), with the difference between the cued and uncued sequence strongest at S4, that is, 20 days post-TMR (p_adj_ = 0.004, Fig. 1d; Supplementary Notes: Main effect of TMR across post-stimulation sessions). Furthermore, there was a main effect of the amount of time post-TMR on cueing benefit (p = 0.046, Fig. 1d), suggesting that the effects of our manipulation develop over time before they emerge at S4 (Rakowska et al., 2024; see Supplementary Notes: Cueing Benefit Across Time). Hence, the relationship between microstructural plasticity across sessions and subsequent cueing benefit at S4 was of particular interest. We also tested for correlations between cueing benefit and percentage of time spent in S2 and S3 sleep stages (Supplementary Table S6).

### 3.1 TMR-related plasticity

We wanted to know whether repeated reactivation of a motor memory trace during a single night could potentially be associated with subsequent plasticity in the brain. Thus, we tested for correlations between changes in brain microstructure within predefined regions of interest (ROIs) over set periods of time and the significant behavioural cueing benefit that emerged at S4. To this end, individual MR parameter maps collected at different sessions were subtracted from each other, yielding measures of early (S1-S2) and late (S2-S3) microstructural plasticity. The resultant difference maps were used as inputs in the joint multi-parameter analysis which aimed to uncover common trends in MD and Fr change measures. Cueing benefit at S4 was entered as the main regressor of interest because we wanted to know whether structural plasticity at earlier timepoints was related to this significant cueing effect observed ~20 days after the manipulation. Notably, we covaried out the non-significant behavioural cueing benefit at S2 and S3 to control for inter-session variations in behavioural trajectories that are unrelated to emergence of the cueing benefit in S4.

We first tested for associations between early plasticity (over the first 24 h e.g. between S1 and S2), and subsequent behavioural benefit at S4. This revealed a positive association between early plasticity in right putamen (34, -6, -8) and cueing benefit at S4 (p = 0.016; Fig. 2a-c, Supplementary Table S1A, Supplementary Fig. S1b). However, this result did not survive correction for multiple ROIs (Supplementary Table S2A), and there was no relationship between cueing benefit and early plasticity in precuneus, hippocampus, or sensorimotor cortex (Supplementary Table S1A). We next tested for associations between plasticity occurring later in the consolidation process (e.g., from 24 h to ~10 days post manipulation) and subsequent behavioural benefit. This revealed a positive relationship between late plasticity in bilateral precuneus (4, -58, 16) and behavioural cueing benefit at S4 when controlling for the behaviour at S2 and S3 (p = 0.027; Fig. 2d-f, Supplementary Table S1Bi-iii, Supplementary Fig. S1a). Late plasticity in left sensorimotor cortex (-60, -18, 14) was also associated with cueing benefit at S4 (p = 0.018; Fig. 2g-i, Supplementary Table S1Biv-vi, Supplementary Fig. S1c). In both cases, greater cueing benefit was associated with greater reductions in MD and greater increases in Fr, and both results survived correction for multiple ROIs (Supplementary Table S2B). No significant clusters were found for the hippocampal or striatal ROI analyses in relation to late microstructural changes (Supplementary Table S1B).

Finally, we performed an exploratory, whole-brain analysis to search for a relationship between cueing benefit and both early and late microstructural plasticity and found no effect in regions outside our pre-defined ROIs (Supplementary Table S1). Together, these results provide evidence for gradual plasticity in the microstructure of precuneus, striatum and sensorimotor cortex that correlates with delayed behavioural effects of TMR. Specifically, our data suggest that TMR benefit 20 days after the manipulation correlates with changes in striatum microstructure over the first 24 h of the consolidation process, while plasticity in precuneus and task-related areas that correlates with this late TMR benefit occurs between 24 h and 10 days of consolidation.

Importantly, we did not have structural MRI data for all participants in all sessions. Thus, our microstructural analyses were limited to just 16 participants for the early delay and 15 participants for the late delay. This small sample size should, therefore, be noted as an important limitation. Additionally, none of the regions where we found correlations between brain plasticity and TMR-related behavioural benefit showed a significant absolute change across either early or late retention intervals when behavioural data were not taken into account.

### 3.2 Individual differences in baseline microstructure

Turning to a separate but closely related issue, we were interested to determine if inter-individual variability in brain microstructure could confer susceptibility to our manipulation. A wide variety of factors are known to influence TMR’s success (Hu et al., 2020). We used baseline (S1) maps of MD and Fr as inputs in the joint multi-parameter analysis, with cueing benefit at S4 entered as a regressor. This showed a relationship between baseline microstructure in right precentral and postcentral gyrus (58, -6, 20) and cueing benefit at S4 (p = 0.008; Fig. 3a-c, Supplementary Table S3; Supplementary Fig. S2), such that individuals with greater response to TMR had less MD and more Fr in this structure at baseline. The result, which survived whole-brain correction (Supplementary Table S4A) and correction for multiple ROIs (Supplementary Table S4B), suggests that the individual variation in sensorimotor microstructure could predict susceptibility to TMR of procedural memory. Importantly, this finding is independent of cueing benefit at S2 and S3, participants’ demographics (sex, age), general sleep patterns (as measured by the Pittsburgh Sleep Quality Index (PSQI) score), time spent in stage 2 of NREM sleep (N2), baseline reaction time, and learning capabilities on the SRTT, all of which were controlled for in the analysis. No correlation with baseline microstructure was revealed within any other pre-defined ROI (Supplementary Table S3).

## 4 Discussion

We set out to investigate the relationship between plasticity in tissue microstructure and beneficial effects of memory reactivation during sleep. To this end, we combined TMR with DW-MRI to test whether baseline micro-architecture of the brain can be used to determine one’s susceptibility to TMR, and whether TMR can impact on brain microstructure, thus giving rise to the behavioural effects observed. First, we find that long-term cueing benefit is associated with gradual microstructural plasticity within memory and task-related regions. Specifically, our data demonstrate that there are early microstructural changes in striatum and late microstructural changes in precuneus and sensorimotor cortex which predict the beneficial effects of cueing 20 days post-TMR. Additionally, we demonstrate that individual differences in baseline sensorimotor microstructure predict long-term behavioural effects of TMR, suggesting that TMR impacts differently on different brains.

Perhaps the most interesting of these results is the relationship between late (24 h – 10 days post-TMR) precuneus plasticity and cueing benefit 20 days post-TMR. This suggests that repeated reactivation of a memory trace during a single night of sleep engenders microstructural changes within precuneus that are associated with the eventual emergence of behavioural benefits from the manipulation. However, these changes do not appear immediately after the manipulation, but need more time and presumably more consolidation to emerge. Functional data collected in parallel with the current dataset have shown that TMR of a procedural memory also engages precuneus functionally, but this occurs relatively early in the consolidation process (Rakowska et al., 2024). Given the well-described role of precuneus in memory retrieval (Treder et al., 2021; Wagner et al., 2005), these results could reflect the difference in recall strength of cued and uncued sequences during their execution. Indeed, TMR and memory reactivation per se share a lot of parallels with memory retrieval. However, the long-term time scale of our current results as well as the microstructural changes that we report suggest that the role of precuneus may extend well beyond immediate retrieval. We speculate that precuneus could build up physical representations of the retrieved information over a longer time-frame. Indeed, precuneus has already been shown to undergo rapid, learning-dependent microstructural plasticity, indicative of memory engram development within this region (Brodt et al., 2018). This structure is known to harbour behaviourally relevant memory representations (Brodt et al., 2016; Jeong & Xu, 2016) and its function is traditionally associated with motor learning (Cohen & Andersen, 2002; Shadmehr & Holcomb, 1997), although it has recently been implicated in declarative memory processing (Brodt et al., 2016, 2018). Notably, the SRTT is not purely procedural, but has a declarative component too (Albouy et al., 2008, 2013). Our observation of plasticity in precuneus during ‘late’ consolidation expands the existing literature by suggesting that microstructural plasticity in this structure as a result of reactivation may continue across several days. Such plasticity could perhaps facilitate the development of a stable and long-lasting memory trace in this structure. This, in turn, gives rise to the emergence of behavioural benefits of reactivation, reflected in the difference between the cued and uncued sequence that we observe 20 days post-TMR. Thus, our data support the idea that (targeted) memory reactivation during sleep has a powerful impact on both memory processing and brain plasticity, and that its effects extend beyond the initial night of sleep.

Our findings also support the suggestion that the long-term cueing benefit of TMR to the SRTT is mediated by the motor system, with early plasticity in putamen and late plasticity in precentral and postcentral gyri. We show that changes within all of these structures are specifically related to the behavioural effects 20 days post-TMR, suggesting that such microstructural plasticity in the motor system may eventually lead to the emergence of behavioural TMR effects. Both striatum and sensorimotor structures are thought to be critical for long-term storage of motor sequences (Doyon et al., 2003). We build on this literature by arguing that memory reactivation during sleep may engender microplasticity within these regions, and thus stabilise memory traces harboured by the cortico-striatal system, shaping the sleep-dependent procedural benefits. Memory reactivation has been observed in ventral striatum immediately after learning (Pennartz et al., 2004) and this could drive the early microstructural plasticity in the adjacent regions, including putamen. In turn, the late microstructural plasticity that we observe in primary motor and somatosensory cortices likely reflects their slowly evolving reorganisation (Ganguly & Carmena, 2009; Gao et al., 2018; Kami et al., 1995; Kleim et al., 2004; Matsuzaka et al., 2007). This late plasticity could also underpin the TMR-related functional engagement and grey matter volume increase of the sensorimotor cortex which we observed in an independent analysis of the current dataset (Rakowska et al., 2024). Interestingly, a recent rodent study found that cortico-striatal functional coupling increases during offline periods of rest and is required for long-term skill learning (Lemke et al., 2021). Furthermore, this coupling seems to be mediated by NREM sleep spindles (Lemke et al., 2021), which are known to be involved in motor learning (Boutin & Doyon, 2020). One intriguing possibility is that neuronal ensembles within sensorimotor cortex and striatum undergo simultaneous replay during post-learning sleep and this leads to their functional coupling. Simultaneous activity in primary motor cortex and dorsal striatum has already been recorded during motor learning (Costa et al., 2004). Our results raise the hypothesis that memory reactivation could also co-occur in these regions during sleep and thereby drive the synaptic plasticity within the underlying substrate. If this is correct, it might suggest that such co-replay could underpin the microstructural changes and the subsequent behavioural benefits observed in our dataset.

Finally, we show that individual differences in the microstructural architecture of sensorimotor cortex measured at baseline are associated with cueing benefit at S4. Our results, therefore, combine to suggest that the microstructure of sensorimotor cortex can both change in response to cueing motor memory reactivation and confer susceptibility to the stimulation. The success of TMR may, therefore, depend on the inter-individual variation in the microstructure of precentral and postcentral gyri. That is, the intrinsic micro-architecture of these task-related regions may either control memory encoding capacity, impact the response to the manipulation, or determine the effectiveness of the reactivation process itself. This finding adds to the existing literature on the factors modulating TMR’s success (Cairney et al., 2016; Creery et al., 2015; Hu et al., 2020; Schechtman et al., 2021), perhaps explaining some of the discrepancies in the TMR literature.

The results of the current study demonstrate that DW-MRI can provide a valuable tool to investigate behaviourally relevant changes in brain microstructure. Furthermore, the multi-parameter approach that we adopted here revealed a common pattern across two diffusion markers: MD and Fr. This not only makes our findings more robust but also provides insights into the biological changes that could underpin sleep-dependent memory consolidation. Biological interpretation of diffusion measures is not straightforward (Zatorre et al., 2012), but combining multiple parameters increases the chances of picking up features that are shared by the two markers. In case of MD and Fr, water diffusion within the restricted (intracellular) volume fraction seems to be a common feature that both markers are sensitive to. Thus, the microstructural changes associated with memory reactivation could involve remodelling of the cylindrical tissue compartments, such as neural and glial cytoplasmic processes (Tavor et al., 2013). Indeed, rapid structural modifications after learning have been reported in astrocytic cytoplasmic processes (Blumenfeld-Katzir et al., 2011; Theodosis et al., 2008) and dendritic spines (Xu et al., 2009). In fact, both NREM (Yang et al., 2014) and REM sleep (Li et al., 2017) have been implicated in dendritic spine plasticity within hours after learning, while disrupting memory reactivation during sleep impaired post-training spine formation (Yang et al., 2014). By the same token, repeated reactivation through TMR during sleep could have boosted similar forms of plasticity in the cylindrical compartments of precuneus, striatum, and sensorimotor cortex in our dataset, thus giving rise to the observed changes in the DW-MRI metrics. However, swelling of cells (particularly astrocytes (Macvicar et al., 2002)), and thus a shift in the ratio of extra- to intra-cellular space (Johansen-Berg et al., 2012; Le Bihan, 2012; Theodosis et al., 2008) could also alter the diffusion properties of the tissue and, consequently, the MD and Fr values. Indeed, synaptogenesis (Kleim et al., 2004) and astrocytic hypertrophy (Kleim et al., 2007) are detectable after motor training and could have contributed to the microstructural plasticity that we report. Nevertheless, the cellular processes driving MD and Fr changes are generally difficult to identify and histological approaches would be needed to confirm the biological interpretation of our results.

In a broader context, DW-MRI allowed us to study the dynamic and distributed nature of the TMR-related changes. Our results show for the first time that microstructural plasticity after cued memory reactivation correlates with cueing-related behavioural advantage. Notably, we show the resulting plasticity encompasses several cortical areas, continues after the stimulation nights, and correlates with long-term consolidation of memories that are reactivated during sleep. Thus, we extend the existing literature by providing direct evidence for reactivation-mediated redistribution of memory traces across the brain (Born et al., 2006; Diekelmann & Born, 2010). Given the long-term character of the detected changes, we speculate that cueing memory reactivation must have either primed the relevant synapses for later processing (Diekelmann & Born, 2010) or biased plasticity-related protein capture towards the targeted memory traces (Seibt & Frank, 2019). This could explain why changes in tissue microstructure over the next 10 days correlated with subsequent behavioural cueing benefit. We further identify precuneus and motor structures as important neocortical memory hubs for long-term retention of procedural memories. The microstructural changes in precuneus, striatum, and sensorimotor cortex were specifically related to the behavioural effects 20 days post-TMR, which was the only time point where we find group-level evidence for the difference between the cued and uncued sequence (Rakowska et al., 2024). This suggests that the microstructural plasticity parallels the gradual development of behavioural cueing benefit, and could eventually contribute to the emergence of behavioural effects of TMR.

## 5 Conclusion

We show that gradual microstructural changes, distributed across several cortical areas, are associated with the emergence of behavioural benefits stemming from memory reactivation. Specifically, we find that microstructural plasticity occurs in precuneus and the motor system and is associated with long-term benefits of procedural memory TMR. These findings support the longstanding belief that stable memory traces develop gradually and reorganise the underlying tissue (Frankland & Bontempi, 2005). Our results are specifically linked to the cueing benefit we observed 20 days post-manipulation. These findings demonstrate that DW-MRI can be used to detect behaviourally relevant microstructural remodelling that underpins sleep-dependent memory consolidation. We also shed new light on the factors that influence TMR’s effectiveness by demonstrating that individual variation in the microstructure of the task-related regions can be used to predict one’s behavioural benefit from the manipulation.

## Supplementary Material

Supplementary Material

## Data Availability

All data collected during the study, scripts that delivered experimental tasks and codes used to conduct the analyses are publicly available at: DOI 10.17605/OSF.IO/B52FV
