# Supplementary material for: Distributed and gradual microstructure changes are associated with the emergence of behavioural benefit from memory reactivation
Source: Imaging Neurosci (Camb). 2025 Aug 22;3:IMAG.a.104. doi: 10.1162/IMAG.a.104 (PMC12375995; doi:10.1162/IMAG.a.104)
Supplement: Supplementary Material [file IMAG.a.104_supp.pdf]

Supplementary Material

Notes

Baseline SRTT performance.

Before sleep, no difference was found between the average reaction time of the cued and uncued sequence for either both hands (BH, $t29 = -0.25$, $p = 0.801$), left hand (LH, $t29 = 0.27$, $p = 0.786$) or right hand (RH, $t29 = -0.50$, $p = 0.621$) (paired-samples t-tests) dataset. Similar results were obtained where comparing random sequences before sleep for all datasets (BH: $z = -0.57$, $p = 0.572$; LH: $z = -0.63$, $p = 0.530$; Wilcoxon signed-rank tests; RH: $t29 = -0.16$, $p = 0.872$; paired-samples t-test). Thus, any post-sleep difference between the sequences can be regarded as the effect of TMR. Published in Rakowska et al. (2024).

Main effect of TMR across post-stimulation sessions.

To test the effect of cueing on the SeqSpecS (either early or late) over time we fitted a linear mixed effects model to our behavioural dataset, with TMR and session entered as fixed effects, and participant entered as a random effect. Inclusion of TMR as a fixed effect improved model fit across all post-stimulation sessions (S2-S4) for late SeqSpecS ($X2(1) = 11.01$, $p = 0.001$; Table S2Aii), but not early SeqSpecS ($X2(1) = 1.55$, $p = 0.214$; Table S2Ai). Thus, the linear mixed effects analysis points to a main effect of TMR on the late SeqSpecS across all post-stimulation sessions. Next, we performed post-hoc comparisons to reveal the session(s) during which late SeqSpecS differed between the two sequences. We found a significant difference between the cued and uncued sequence performance at S4 (20 days post-stimulation, $padj = 0.004$) but not at S2 (24 h post-stimulation, $padj = 0.282$) or S3 (10 days post-stimulation, $padj = 0.282$) (Table S4A, Fig.2B). Together, these findings point to a main effect of TMR across all post-stimulation sessions, with the difference between the cued and uncued sequence strongest 20 days post-TMR. Published in Rakowska et al. (2024).

Cueing Benefit Across Time.

To explore how the TMR effect evolves over time, we used late SeqSpecS, as in prior studies (Rakowska et al., 2021; Cousins et al., 2014). Specifically, we calculated the difference between late SeqSpecS of the cued and uncued sequence for each session, and refer to this as the (late) cueing benefit. Next, we used a linear mixed effects analysis to determine if cueing benefit changes across post-stimulation time. Inclusion of the number of days post-TMR as the fixed effect improved model fit on the extent of cueing benefit ($\chi2(2) = 3.97$, $p = 0.046$; Fig.2C; Table S5A), suggesting that the effects of TMR may develop in a gradual time-dependent manner. Published in Rakowska et al. (2024).

Tables

Table S1. Microstructural plasticity associated with cueing benefit at different sessions.

Cluster statistics for precuneus, putamen and sensorimotor cortex which showed a positive relationship between microstructural plasticity (MD and Fr) and cueing benefit at different sessions. MD and Fr were either analysed together in a multi-parameter framework (i, iv) or separately using uni-parameter analyses (ii-iii, v-vi).

**Analysis**	**ROI**	**Region**	**MNI x, y, z (mm)**	**Number of voxels**	**T peak**	**P_{FWE} peak**
A. [Early microstructural plasticity * cueing benefit at S4]						
i) Multi-parameter	Dorsal Striatum	Right putamen	34, -6, -8	633	-	**0.016***
ii) Uni-parameter: MD		Right putamen	32, 2, -10	563	5.20	0.054^
iii) Uni-parameter: Fr		-	20, 8, -4	79	3.34	0.563
B. [Late microstructural plasticity * cueing benefit at S4]						
i) Multi-parameter	Precuneus	Right precuneus	4, -58, 16	1943	-	**0.027***
ii) Uni-parameter: MD		Right precuneus	-6, -54, 30	1992	10.08	**0.033***
iii) Uni-parameter: Fr		-	8, -52, 6	867	8.93	0.116
iv) Multi-parameter	Sensorimotor Cortex	Left precentral and postcentral gyri	-60, -18, 14	2159	-	**0.018***

v) Uni-parameter: MD		Left precentral and postcentral gyri	-60, -20, 14	3987	13.15	**0.010***
vi) Uni-parameter: Fr		-	48, -8, 56	54	7.71	0.783

*Regions listed were significant at peak voxel threshold of $p_{FWE} < 0.05$, after correction for multiple voxel-wise comparisons within pre-defined bilateral ROI (as listed in the first column) and the number of modalities (two modalities, MD and Fr). Peak voxel MNI coordinates and peak T values are given. Covariates of no interest included in the analysis: age, sex, PSQI score, baseline reaction time, baseline learning capabilities on the SRTT, cueing benefit at S2 and at S3. S3-4: Session 3-4; * $p < 0.05$, ^ $p < 0.06$. n = 16 for (A), n = 15 for (B).*

Table S2. Multiple ROIs correction for the correlational results reported in Fig. 2.

Results of the multi-parameter analyses performed on each correlational result, correcting for all the ROIs.

**Region**	**MNI x, y, z (mm)**	**Number of voxels**	**P_{FWE} peak**
[Early microstructural plasticity * cueing benefit at S4]			
Right putamen	34, -6, -8	633	0.194
[Late microstructural plasticity * cueing benefit at S4]			
Right precuneus	-60, -18, 14	4110	**0.015***

Regions listed were significant at peak voxel threshold of $p_{FWE} < 0.05$, after correction for multiple comparisons within a single mask combining all the pre-defined ROIs: (1) sensorimotor cortex, (2) hippocampus and parahippocampus, (3) dorsal striatum, (4) precuneus. Peak voxel MNI coordinates are given. Note that the significant cluster in (B) contains both bilateral precuneus (peak MNI coordinates shown) and left precentral and postcentral gyrus. n = 16 for (A), n = 15 for (B).

Table S3. Baseline microstructure and TMR susceptibility.

Cluster statistics for sensorimotor cortex which showed a positive relationship between baseline microstructure and cueing benefit at S4. n = 16.

**Analysis**	**ROI**	**Region**	**MNI x, y, z (mm)**	**Number of voxels**	**T peak**	**P_{FWE} peak**
i) Multi-parameter	Sensorimotor Cortex	Right precentral and postcentral gyri	62, -4, 16	2916	-	**0.008***
ii) Uni-parameter: MD		-	60, 6, 16	592895	17.37	**0.021***
iii) Uni-parameter: Fr		-	32, -36, 38	592895	10.89	0.092

Table S4. Whole brain and multiple ROIs correction for baseline microstructure and TMR susceptibility results.

Results of the multi-parameter analyses performed on the correlational results reported in Fig.3, correcting for whole brain grey matter and all the pre-defined ROIs.

**ROI**	**Region**	**MNI x, y, z (mm)**	**Number of voxels**	**P_{FWE} peak**
A. Whole brain grey matter	Left Cerebellum	-4, -72, -44	9871	**0.039***
B. 4 pre-defined ROIs	Right Precentral gyrus	62, -4, 16	2916	**0.011***

Regions listed were significant at peak voxel threshold of $p_{FWE} < 0.05$, after correction for multiple comparisons within (A) whole brain grey matter and (B) a single mask combining all the pre-defined ROIs: (1) sensorimotor cortex, (2) hippocampus and parahippocampus, (3) dorsal striatum, (4) precuneus. Peak voxel MNI coordinates are given. Note that the significant cluster in (A) contains right precentral and postcentral gyri (peak MNI coordinates shown). n = 16.

Table S5. Sleep parameters.

Total recording duration, total sleep time, time spent in each sleep stage and time scored as movement presented as average (minutes ± SEM) and as percentage of the total recording duration. Total sleep time was calculated by subtracting the time spent awake from the total recording duration. N1-N3: stage 1 – stage 3 of NREM sleep. REM: Rapid Eye Movement sleep. n = 29.

	Percentage of total recording duration [%]	Mean duration ± SEM [min]
Total recording duration	100%	524.19 ± 10.29
Total sleep time	88.38%	463.29 ± 12.89
Wake	11.50%	60.90 ± 10.37
N1	4.52%	23.33 ± 1.73
N2	46.35%	242.45 ± 8.41
N3	19.91%	104.22 ± 4.05
REM	15.86%	83.43 ± 4.30
Movement	1.61%	8.43 ± 1.32

Table S6. Cueing benefit and the duration of N2 and N3.

*Results of Pearson's correlations between cueing benefit and the percentage of time spent in N2 and N3. Both the uncorrected and FDR corrected p-values are reported. df: degrees of freedom; S2-4: Session 2-4; SeqSpecS: Sequence Specific Skill; N2-3: Stage 2-3 of NREM sleep. *p < 0.05.*

	**Time spent in N2 [%]**				**Time spent in N3 [%]**			
	df	Pearson's correlation	p-value	p-value (FDR corr)	df	Pearson's correlation	p-value	p-value (FDR corr)
S2	25	0.197	0.324	0.324	27	-0.015	0.939	0.939
S3	20	0.378	0.082	0.144	21	-0.031	0.887	0.939
S4	19	0.372	0.096	0.144	21	0.089	0.697	0.939

Figures

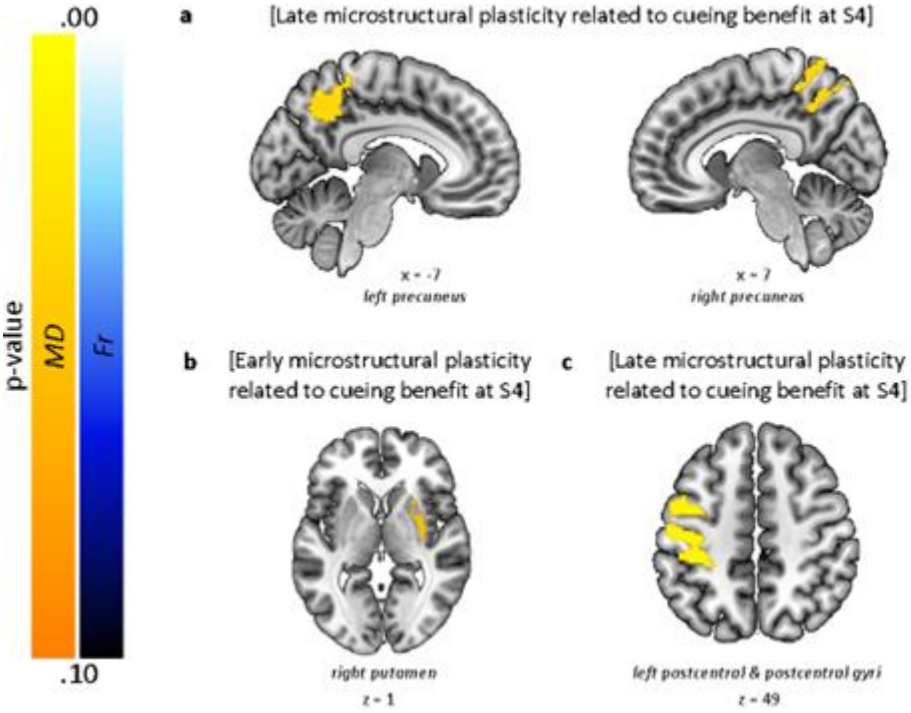

Fig. S1. Results of uni-parameter analyses conducted for MD and Fr separately, testing the same relationships as in Fig.2. *(a-c) Uni-parameter results for the same contrasts as in Fig.2. Colour bars indicate p-values at* $p_{FWE} < 0.1$*, corrected for multiple modalities and the number of voxels within the chosen ROI. In orange, MD clusters; in blue, Fr clusters. Results are overlaid on a Montreal Neurological Institute (MNI) brain. Colour bars indicate 1 - p-value, derived from 5000 permutations. S1-4: Session 1-4. n = 15 for (a, c), n = 16 for (b).*

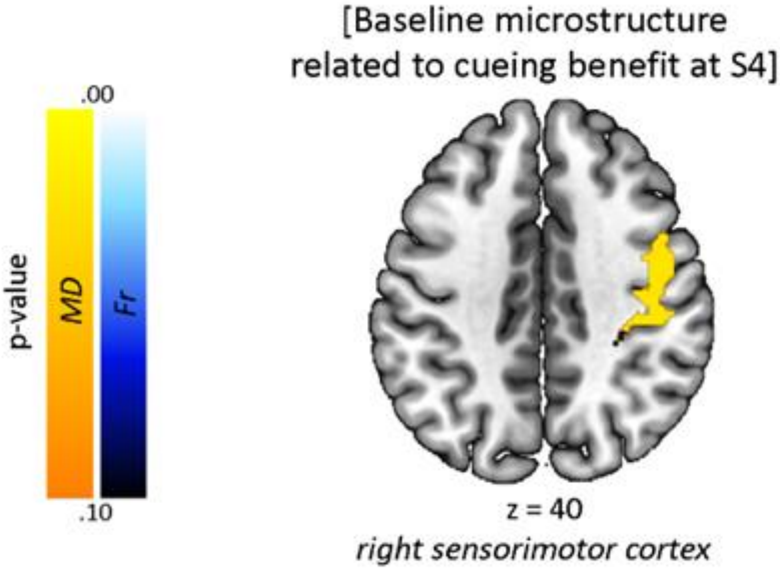

Fig. S2. Results of uni-parameter analyses conducted for MD and Fr separately, testing the same contrast as in Fig.3*. Colour bars indicate p-values at $p_{FWE} < 0.1$, corrected for multiple modalities and the number of voxels within the chosen ROI. In orange, MD clusters; in blue, Fr clusters. No Fr clusters above the $p_{FWE} < 0.1$ threshold were revealed. Results are overlaid on a Montreal Neurological Institute (MNI) brain. Colour bars indicate 1 - p-value, derived from 5000 permutations. S1-4: Session 1-4. n = 16.*

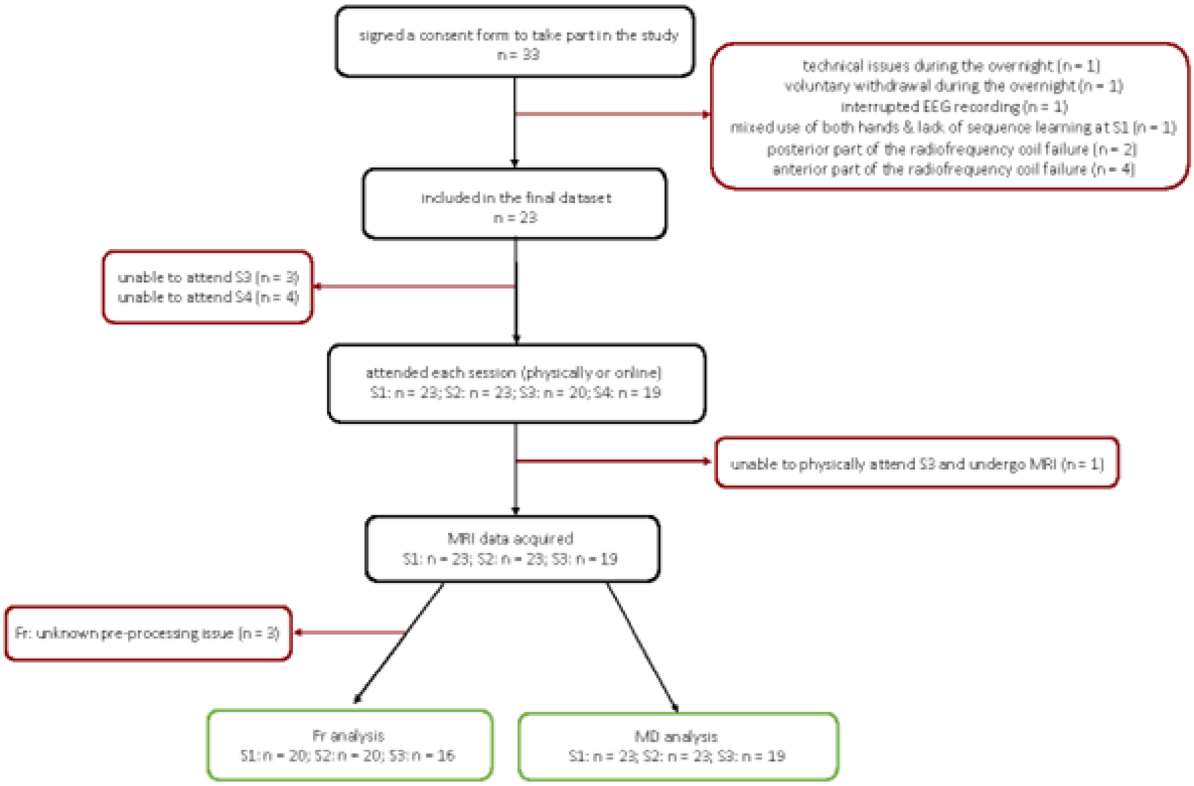

Fig. S3. A flowchart showing participants included and excluded from the analysis. *In black and white, the number of participants included in the study at different time points, with the final sample size shown in green. In red, the number of participants excluded from different analyses, together with a reason for the exclusion. S1-S4: Session 1 – Session 4; Fr: Restricted water fraction; MD: Mean Diffusivity.*

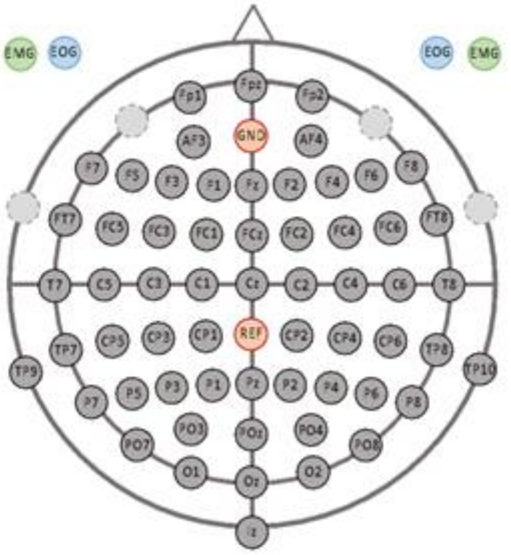

***Fig. S4. EEG electrodes layout**. Orange: ground (GND) and reference (REF) electrodes; light grey (dashed circles): original position of the electrodes used to record EMG and EOG; green: EMG electrodes; blue: EOG electrodes.*
